# Validation of an LC-MS/MS Method for the Quantification of Caffeine and Theobromine Using Non-Matched Matrix Calibration Curve

**DOI:** 10.3390/molecules24162863

**Published:** 2019-08-07

**Authors:** Vera M. Mendes, Margarida Coelho, Angelo R. Tomé, Rodrigo A. Cunha, Bruno Manadas

**Affiliations:** 1 CNC-Center for Neuroscience and Cell Biology, University of Coimbra, 3004-504 Coimbra, Portugal; 2 Chemistry Department, Faculty of Sciences and Technology, University of Coimbra, 3004-535 Coimbra, Portugal; 3 Department of Life Sciences, Faculty of Sciences and Technology, University of Coimbra, 3000-456 Coimbra, Portugal; 4 Faculty of Medicine, University of Coimbra, 3004-504 Coimbra, Portugal

**Keywords:** caffeine, LC-MS/MS, non-matched matrix calibration curve

## Abstract

Caffeine is one of the most widely consumed psycho-stimulants. The study of the beneficial effects of caffeine consumption to decrease the risk of developing several neuropsychiatric pathologies is receiving increasing attention. Thus, accurate and sensitive methods have been developed, mainly by LC-MS/MS, in order to quantify caffeine and its metabolites. These quantifications of caffeine and its metabolites by LC-MS/MS require a considerable effort to select or find a surrogate matrix, without the compounds of interest, to be used in the calibration curves. Thus, we evaluated the possibility of using calibration curves prepared in solvent instead of calibration curves prepared in human plasma. Results show that the calibration curves prepared in solvent and in human plasma were similar by comparing their slopes and interceptions, and the accuracy and precision were within the limits of acceptance for both calibration curves. This work demonstrates that, by using internal standards, it is possible to use a calibration curve in solvent instead of a calibration curve in plasma to perform an accurate and precise quantification of caffeine and theobromine.

## 1. Introduction

Caffeine is one of the most widely consumed psychoactive substance and can be found in several beverages and foods like coffee, tea, cola drinks, chocolate products and in some cases medication [1]. Daily consumption can vary substantially between each individual or population depending on their genetic predisposition and their dietary habits and caffeine intake, and absorption and metabolism can be influenced by several exogenous and endogenous factors [2]. A population-based study (37,602 consumers with age ≥2) estimated the caffeine intakes from beverages and concluded that coffee (64%) followed by tea (17%) and energy drinks (17%) were the major sources accounting for 98% of the daily caffeine intake [3]. In line with the concern of knowing if caffeine brings negative health effects, there are indications that habitual caffeine consumption of up to 400 mg per day is not a risk for potential adverse effects [4,5,6]. For children and adolescents, caffeine consumption up to 3 mg/kg bw per day was considered safe but for pregnant women, the maximum was of 200 mg/day of caffeine daily intake [5]. Caffeine was also a target of study for its potential as a drug of dependence, but it was concluded that the relative risk of addiction of caffeine is low even though it fulfills some of the criteria of drug dependence for the doses usually consumed by the population in general [7]. Curiously, until 2004 the International Olympic Committee listed caffeine as a prohibited substance, but due to the difficulty in distinguishing performance-enhancing doses from daily caffeine intake, it is now only included in the Monitoring Program list from the World Anti-Doping Agency (WADA) [8,9]. In spite of some purported negative effects of caffeine, the bulk of the available evidence shows that a moderate consumption of caffeine has a positive correlation with a lower risk of developing neurodegenerative disorders, such as Alzheimer’s disease [10,11] and Parkinson´s disease [12], as well as the incidence of mood-related disorders such as major depression [12] and suicide [13]. 

The effects on the central nervous system (CNS), considering low to moderate caffeine consumption, have been extensively reviewed and it is generally accepted that caffeine acts as an antagonist of adenosine receptors causing an ergogenic effect as well as a normalization of information processing in neuronal networks [13,14,15,16,17]. Other biochemical mechanisms of action for caffeine have been described as the inhibition of phosphodiesterases (PDEs), the release of intracellular calcium and interference with GABA-A receptors, but these are associated with the toxic effects caused by higher doses of caffeine [16,17]. 

Chemically, caffeine (1,3,7-trimethylxanthine) is as methyl-xanthine, which is rapidly absorbed by the gastrointestinal tract and it is metabolized in the liver to form three major metabolites: 84% paraxanthine (1,7-dimethylxanthine), 12% theobromine (3,7-dimethylxanthine), and 4% theophylline (1,3-dimethylxanthine) [2,18,19]. There are some indications that theophylline and paraxanthine are more potent inhibitors of adenosine receptors than the parent caffeine [20,21]. Thus, the simultaneous measurement of caffeine and their main metabolites should be considered in analytical quantitation methods. There is an increasing interest in studying the health effects of methylxanthines and the biological processes involved in ageing-associated diseases [22,23], namely several pathologies such as cardiovascular diseases [22,23,24], neurodegenerative disorders [11,12,25,26,27,28], diabetes [29,30] and cancer [31,32]. An accurate and sensitive technique is usually important to measure these compounds. Liquid chromatography coupled to mass spectrometry (LC-MS) instrumentation has been a powerful tool for the quantification of compounds in human body tissues and fluids mainly due to the high selectivity combined with the high sensitivity of the technique. 

There are several methods described in the literature for the simultaneous absolute quantification of caffeine and their main three metabolites in plasma [18,33,34,35]. The sample preparation is generally very similar involving a protein precipitation step using an organic solvent (methanol) and in some cases adding a filtration or solid-phase extraction (SPE) step before the LC-MS analysis. The use of stable isotope internal standards is essential for LC-MS absolute quantification because they can improve the reproducibility between injections, adjust the loss of sensitivity during a running batch of samples and account for matrix effects that can occur during the ionization process [36,37]. Calibration curves also need particular attention and it is always preferred to prepare them in the correspondent matrix of analysis due to the possible matrix effects caused by co-eluting matrix components. An analytical problem arises when the quantification involves endogenous compounds where it is important to select an appropriate surrogate matrix which is a matrix whose composition is identical to the analyzed samples but with the absence of the analyte [38]. Although caffeine and its metabolites are exogenous compounds, the same challenge of obtaining human plasma without the compounds of interest occurs during the method validation process. There are some matrices already validated for caffeine quantification which were used to prepare synthetic plasma without caffeine [39], and fetal bovine serum matrix [34]. However, none of these matrices truly represents the original matrix and finding caffeine-free human plasma volunteers can be challenging. 

For this reason, the present work studies the possibility of using calibration curves prepared in solvent instead of calibration curves prepared in a human plasma matrix. The purpose of the study was to take advantage of the use of the internal standards to compensate for losses during sample preparation and possible matrix effects during ionization. 

## 2. Results and Discussion

### 2.1. Optimization of LC-MS Conditions 

**Figure 1:**
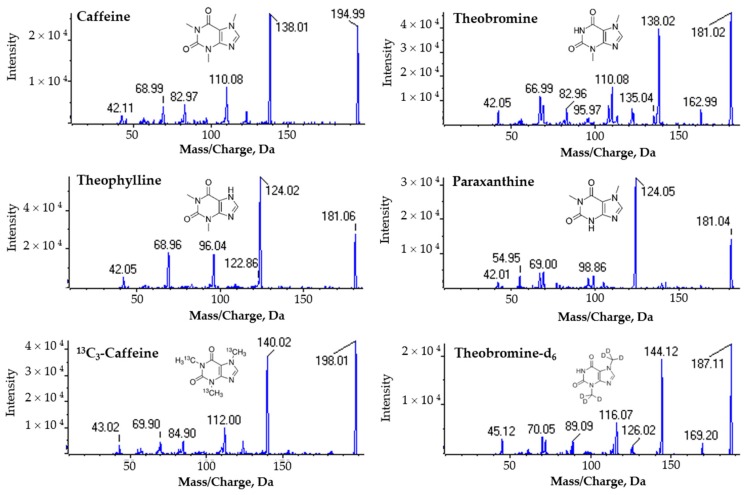
Fragmentation mass spectra by collision-induced dissociation (CID) of caffeine, theobromine, theophylline, paraxanthine and the internal standards ^13^C_3_-caffeine and theobromine-d_6_. Standard solutions were directly infused into the mass spectrometer in positive ionization mode and fragmentation was performed with a collision energy ramping. The presented fragmentation mass spectra are the combination of fragmentation data acquired for collision energies between 20–45 eV.

Compound optimization was performed in positive ionization mode through the acquisition of fragmentation mass spectra in order to support the selection of the appropriate multiple reaction monitoring (MRM) transitions for each compound (Figure 1). Each molecule presented a predominant intense fragment which was further considered for the MRM method acquisition. Caffeine was monitored by the transition 195/138, theobromine by 181/138, and theophylline and paraxanthine by the shared transition 181/124.

The chromatography was optimized for reverse phase mode (C18 column) and a low separation efficiency was observed between the chromatographic peaks corresponding to theophylline and paraxanthine. Consequently, a new set of tests were performed in order to improve separation using different stationary phases (Amino and Polar-RP), without further improvement in the separation (data not shown). Afterward, the manual re-analysis of the fragmentation mass spectrum was performed in order to find fragments that could be specific for each compound. Although some specific fragments were detected, the sensitivity was insufficient, and the shared transition 181/124 was selected for further validation of the MRM method. 

### 2.2. Method Validation 

#### 2.2.1. Selectivity

**Figure 2:**
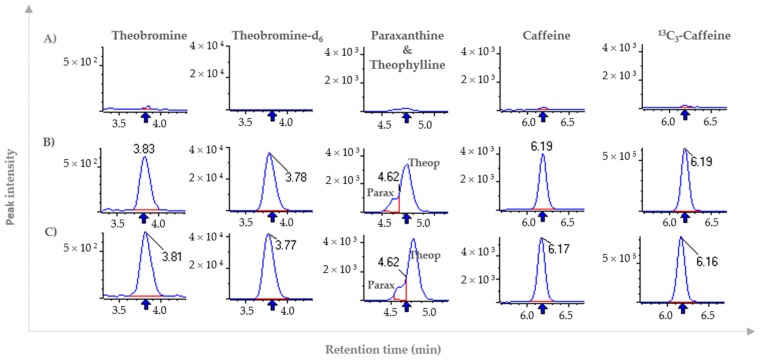
Extracted ion chromatograms corresponding to caffeine, theobromine, theophylline, paraxanthine and internal standards ^13^C_3_-caffeine and theobromine-d_6_ in (**A**) “blank” plasma samples, in (**B**) “blank” plasma spiked with each molecule and (**C**) pure solutions of each compound.

Selectivity was evaluated analyzing six human plasma samples as blanks and spiked with known amounts of each molecule, including the internal standards. Results show that no interfering peaks were detected for the transitions analyzed by MRM (Figure 2). The only interference detected was between theophylline and paraxanthine.

In order to assess the possible contamination due to the overlap during peak area integration for paraxanthine and theophylline, preliminary experiments were performed by preparing calibration curves containing both molecules and one calibration curve for each compound separately. Comparing the results from the calibration curves of the two compounds prepared in the same mixture or separately, the peak areas integration was similar for both situations (Figure S1). Although, selectivity was acceptable only for caffeine and theobromine, further parameters included in the method validation were also evaluated for theophylline and paraxanthine.

#### 2.2.2. Linearity, Limits of Detection and Quantification

The homoscedasticity was evaluated for all compounds and results show that the residuals were not homogenously distributed and the dispersion was higher for the highest concentrated point suggesting that data are heteroscedastic for all molecules (Figure S2). Consequently, the best weighting factor was determined and, for all molecules, the weighting factor 1/x^2^ was the one that reproduced the least sum of the relative errors (%RE) providing the most adequate approximation of variance either for solvent or plasma calibration curves (Tables S1 and S2). In fact, previous studies showed that 1/x^2^ should be used for all LC-MS/MS bioanalytical assays [40]. 

The linearity of the method was evaluated for the calibration curves prepared in plasma and solvent for the five non-consecutive days (Table 1). By applying the weighting factor 1/x^2^, the relative errors (%RE) for each calibrant were below the limits of acceptance, ≤20% for the first calibrant and ≤ 15% for other calibrants (Figure S3). For each molecule, the results for the regression parameters (slope, Y-intercept, and coefficient of determination - R^2^) were determined and are shown in Table 1, represented by mean ± SD considering solvent and plasma preparation. The coefficient of determination (R^2^) was acceptable (>0.99) for all molecules and for both matrices. Results for the limit of detection (LOD) and limit of quantification (LOQ) show that the limits were similar in plasma and solvent (Table 1).

With the purpose of using calibration curves prepared in solvent to avoid the use of calibration curves prepared in plasma, the similarity was evaluated for both regression methods (solvent and plasma). For all the compounds, the slope and Y-Intercept did not show significant statistical differences (95% confidence level) between calibration curves prepared in plasma (*n* = 5) compared to calibration curves prepared in solvent (*n* = 5) (Table 1). 

**Figure 3:**
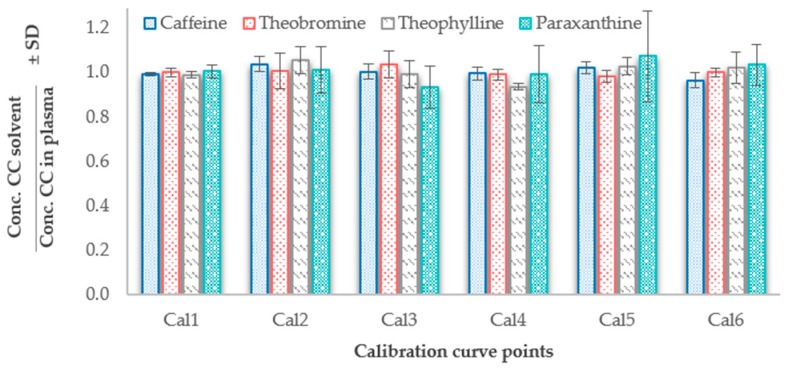
Comparison of the regressed calculated concentrations (conc.) using the calibration curve (CC) prepared in plasma compared to the calibration curve prepared in solvent. The bars represent each one the six calibrators showing the ratios of the calculated concentrations using the solvent regression model against the plasma regression model. For each calibrator point (bar), the mean ± SD was calculated using data acquired from five non-consecutive days (*n* = 5).

The concentrations of the calibrators from the calibration curves were re-calculated using the equations of the regression methods using the weighting factor 1/X^2^. Consequently, the ratio between the regressed concentrations in solvent and plasma calibration curves were determined and it was observed that data from both matrices were in accordance (ratio close to 1) for all the calibration points (Figure 3). The mean of the concentration ratios of all analytes was between 0.93 and 1.07. The coefficient of variation (%CV) was acceptable for caffeine, theobromine, and theophylline (0.8–8%) but paraxanthine suffered from higher standard deviations compared to the other molecules resulting in a range of CV% between 3–19% (Table S3). 

#### 2.2.3. Precision and Accuracy

**Figure 4:**
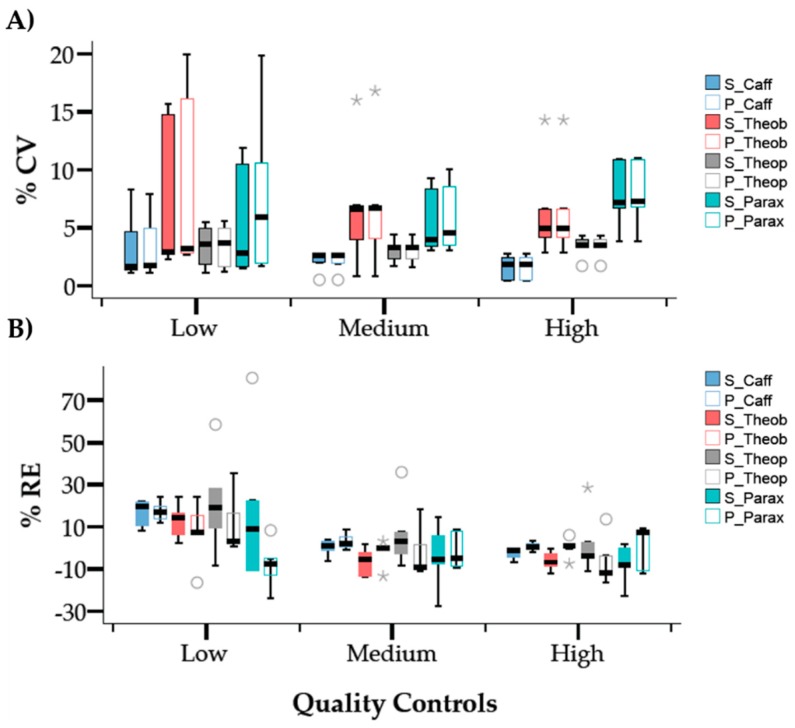
Box-plot representation for the results of the intra-day (*n* = 3) (**A**) precision and (**B**) accuracy for caffeine, theobromine, theophylline, and paraxanthine determined using calibration curves prepared in solvent (S) and plasma (P). Results for the precision study are expressed as the percentage of coefficient of variation (%CV) and for accuracy as the percentage of relative error (%RE). Each box plot represents determinations for five days. The circles and asterisks are outliers that represent cases having values more than 1.5 or three times the height of the boxes (interquartile difference Q3-Q1), respectively.

In order to evaluate the precision and accuracy of the quantification of the four molecules, concentration values were re-calculated for the three levels of the quality controls (QC) using calibration curves prepared in solvent and in plasma in five non-consecutive days. The intra-day precision and accuracy results are summarized in Figure 4 with the coefficient of variation (CV%) and relative error (RE%), respectively. The results show that the quantification method suffered from data dispersion in the lower range of concentrations however, the CV% values were below the acceptable limit (<20%). For all molecules analyzed, the precision results were very similar when comparing the quantification using the calibration curve in solvent with the quantification using the calibration curve in plasma. In addition, data also shows higher precision for caffeine and theophylline quantification demonstrated by the smallest interquartile range. The degree of closeness of the measured concentration value to the known “true” concentration value was evaluated by determining the percentage of the relative error (%RE). Despite some outliers detected during the analysis and represented in the boxplot graphic (Figure 4B), the quantification was accurate using both calibration curves (plasma and solvent) even for the low-level QC where the quantification suffered a slight overestimation.

The inter-day precision and accuracy were evaluated for five non-consecutive days after quantifying the four analytes in the quality control samples using calibration curves prepared in solvent and in plasma (Table 2). The results for the precision (%CV) were nearly the same comparing the quantification performed in both calibration curves and the coefficient of variation values ranged between 1.6–8.4%. When comparing the inter-day accuracy obtained using each one of the calibration curves (plasma and solvent) results were also very similar. However, for the lowest QC standards higher RE were observed in solvent, and for theophylline the limit of acceptance was slightly exceeded by 1%.

#### 2.2.4. Extraction Efficiency and Matrix Effect

The extraction efficiency (recovery) was evaluated by comparing the peak areas of each molecule from the quality control samples (low, medium and high concentration levels) spiked before and after the extraction procedure (Table 3). The matrix effect (ionization suppression/enhancement) was also evaluated for the three levels of quality controls but comparing peak areas of each molecule spiked after the extraction procedure with the peak areas in a pure solution. The extraction efficiency of caffeine was 73–79% and the extraction efficiency of the internal standard (^13^C_3_-caffeine) was also very similar (approximately 78%). For theobromine and theophylline, recoveries were similar (84–91%) and the internal standard (theobromine-d_6_) also had a recovery of around 89%. The coefficients of variation were acceptable but, as commonly observed, higher for the low concentration measurements. The recovery results for paraxanthine were revealed to be less reproducible (higher CV values) for the medium and high concentration levels which can explain the higher recovery percentage values in these concentration levels (86–98%). It might be due to the integration step during data processing and not due to the extraction protocol for plasma samples. Matrix effect results were satisfactory for all molecules and they suggest that they do not influence the analysis of plasma samples by electrospray ionization in positive mode. Chen et al. (2017) studied the influence of matrix effects and showed that the signal suppression was high for paraxanthine, theobromine and theophylline considering different concentrations of formic acid in the mobile phase [18], however, our results did not show signal suppression considering the same formic acid concentration.

The absence of matrix effects can be indicative that the calibration curves prepared in plasma and in solvent can equally be used to perform the absolute quantification. The only drawback can be related to the obtained extraction efficiencies below 100%, meaning that performing absolute quantification using the calibration curve in solvent could yield underestimated results. However, this was resolved by the use of internal standards which equalized the regression methods from the two different matrices (Figure S4). To strengthen this argument, the results of the accuracy obtained for the two matrices were similar (Figure 3 and Table 2).

#### 2.2.5. Carryover

**Figure 5:**
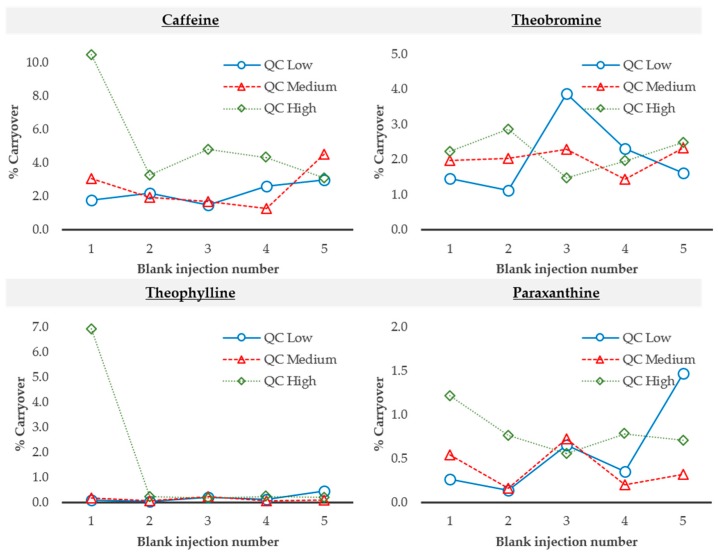
Carryover results for caffeine, theobromine, theophylline, and paraxanthine. After the injection of each concentration level of the quality controls, five blank solvent samples were analyzed and peak areas were divided by the peak area obtained for the LOQ. The graphs show the percentage of this ratio for each one of the five injections and considering each analyte. QC—quality control.

Carryover was evaluated in order to detect possible contamination of the molecules between each LC-MS analysis and to avoid having a potential source of analytical error in the quantification. To study this parameter, five blank solvent samples were analyzed after the injection of each quality control level (low, medium and high). Results show that carryover was always below 10% (Figure 5), which is below the recommended threshold (<20% of LOQ). 

### 2.3. Applicability of the Method in Human Plasma Samples

The LC-MS/MS quantification methodology described in this paper was tested in human plasma samples for caffeine consumption varying from 0 to 100 mg/day and theobromine consumption varying between 0 to 75 mg/day (Table 4). In samples 1 and 2, where no consumption or a very low amount of caffeine and theobromine was ingested, no caffeine or other metabolite was quantified with the exception of paraxanthine in sample 1. When caffeine consumption was present, it was possible to quantify caffeine in plasma as well as its metabolites and for the majority of samples paraxanthine had the highest values and theophylline the lowest values for quantification. This result was expected since it is known that caffeine metabolizes to paraxanthine in a percentage of 84% and theophylline is the less abundant metabolite (4%). There are some results lacking consistency, for example, sample 6, where no caffeine and theophylline were quantified when the initial caffeine consumption was around 50 mg/mL. These results suggest inter-individual differences in metabolizing caffeine and their metabolites and that there are certainly other factors that should be taken into account in these types of studies. 

## 3. Materials and Methods 

### 3.1. Chemicals

Caffeine (1 mg/mL), theobromine (0.1 mg/mL), theophylline (1 mg/mL), paraxanthine (1 mg/mL), ^13^C_3_-caffeine (1 mg/mL) stock solutions and theobromine-d_6_ (98% purity) were purchased from Sigma. Acetonitrile, methanol, and water were LC-MS grade and were from VWR. Formic acid was LC-MS grade and purchased from Amresco. 

### 3.2. Compound Optimization for the MRM Acquisition Method

For the optimization of the MRM acquisition method, direct infusion was performed into the mass spectrometer and automatic compound optimization was performed for each molecule, including the internal standards. For the automatic compound optimization, a full scan mass spectrum was acquired varying the declustering potential (DP) from 0 to 400 V followed by fragmentation by CID for the collision energy range from 5 to 130 eV.

### 3.3. Liquid Chromatography-Mass Spectrometry Instrumentation

Samples were analyzed on an LC Nexera system (Shimadzu) coupled to a hybrid triple quadrupole/linear ion-trap 4000 QTrap mass spectrometer operated by Analyst 1.6.1 (Sciex). The injector was a CTC-xt (PAL System). The chromatographic separation was performed using the 3 μm Gemini C18 column (50 × 2.0 mm, 110Å, Phenomenex) with a 4 × 2.0 mm C18 guard-column (Phenomenex). The flow rate was set to 250 µL/min and mobile phases A and B were 0.1% formic acid in water and 0.1% formic acid in acetonitrile, respectively. The LC program consisted in: 2% of B (0–0.3 min), 2–10% of B (0.3–5.0 min), 10–90% of B (5.0–6.0 min), 90% of B (6.0–7.0 min), 90–2% of B (7.0–7.1 min) and 2% of B (7.1–9.0 min). The ionization source (ESI Turbo V) was operated in the positive mode set to an ion spray voltage of 5500 V, 35 psi for nebulizer gas 1 (GS1), 20 psi for the nebulizer gas 2 (GS2), 30 psi for the curtain gas (CUR), and the temperature was 450 °C. All molecules were analyzed by scheduled multiple reaction monitoring (sMRM) setting Q1 and Q3 at unit resolution, the entrance potential (EP) at 10 eV, the collision cell exit potential (CXP) at 15 eV and the collision gas (CAD) was at 8 psi. The MRM detection window was set to 60 s and the target scan time to 1 s. The MRM transitions for each compound and the parameters used are shown in Table 5.

### 3.4. Method Validation

Method validation was evaluated for selectivity, linearity, limits of detection and quantification, precision and accuracy, extraction efficiency, matrix effect, and carryover. 

#### 3.4.1. Selectivity

The selectivity of the method was evaluated by comparing chromatograms of blank human plasma from six different individuals and blank human plasma spiked with caffeine, theobromine, theophylline, paraxanthine, and the internal standards.

#### 3.4.2. Linearity and Limits of Detection and Quantification

The study of the linearity was performed for calibration curves prepared in solvent and prepared in “blank” human plasma for five different days. Calibration curves in solvent were prepared by successive dilution steps to give six calibration points for each compound. For caffeine, the calibrants concentrations used were 3.9, 13.6, 58.5, 174.8, 388.6 and 582.6 ng/mL. For theobromine, theophylline, and paraxanthine the calibrant concentrations were 3.6, 12.6, 54.2, 162.2, 360.5 and 540.5 ng/mL. The final concentrations of the internal standards were 39.6 ng/mL for ^13^C_3_-caffeine and 36.0 ng/mL for theobromine-d_6_.

Calibration curves in plasma were prepared by spiking 50 µL of “blank” human plasma with each calibrator prepared in solvent and the internal standards before protein precipitation with methanol. The following sample preparation steps were the same as described in Section 3.5 (“Plasma sample preparation”). 

The limit of detection (LOD) and the limit of quantification (LOQ) were determined in solvent and plasma based on the linear regression data as:

LOD = 3S_a_/b (1)

LOQ = 10Sa/b(2)

where S_a_ is the standard deviation of the regression line and b is the slope of the calibration curves.

The homoscedasticity assumption was tested for the linear regression analysis by plotting the residuals compared to the lowest and the highest concentrations considered for the calibration range [41]. The best weighting factor was determined based on the lowest value of the ∑ |%RE| and acceptable R^2^ (≥0.99). The regression parameters slope and Y-intercept were tested using the independent samples Student´s t-test for 95% confidence. 

#### 3.4.3. Intra and Inter-Day Precision and Accuracy

Intra-day precision and accuracy evaluation were based on the QC sample replicates from the same day and inter-day precision and accuracy based on QC samples from five days for each concentration level. The QC samples were prepared in solvent and plasma at three levels of concentration on five different days. The concentration levels (low, medium and high) for caffeine were 5.9, 38.8 and 340.2 ng/mL and for theobromine, theophylline and paraxanthine were 5.5, 36.0 and 315.7 ng/mL, respectively.

#### 3.4.4. Extraction Efficiency and Matrix Effect

The recovery of each compound was determined for the three levels of concentration by comparing the responses of QC samples prepared in plasma with the responses of post-extracted “blank” human plasma spiked at equivalent concentrations. The matrix effect was determined for the same concentration levels by comparing responses of the spiked post-extracted “blank” human plasma with the responses of each compound in pure solution. 

#### 3.4.5. Carryover

Carryover was evaluated by analyzing five different solvent blank samples after the injection of QC samples for the three levels of concentration. The percentage of carryover was determined by dividing the peak areas of each blank by the peak areas obtained for the LOQ of each analyte.

### 3.5. Plasma Samples 

Collection of plasma was performed at the participating centers according to the recommendations of BIOMARKAPD [42]. Blood was collected in EDTA tubes, centrifuged according to routine local protocols, and plasma aliquots were frozen at –80 °C.

### 3.6. Plasma Samples Preparation

Plasma samples (50 µL) were spiked with 50 µL of the internal standards solution (^13^C_3_-caffeine at 79.2 ng/mL and theobromine-d_6_ at 72 ng/mL) and protein precipitation was performed using methanol (three volumes), followed by centrifugation at 14,000× *g* for 20 min. The supernatant was collected, evaporated in a vacuum centrifuge concentrator and samples were resuspended in 100 µL of 2% ACN + 0.1% FA and sonicated for 2 min (Sonics, 750W, cup-horn, Pulse: 1 sec; Pause: 1 sec, Amplitude 40%). Samples were centrifuged for 5 min at 14,000× g and the volume of injection was 10 µL.

## 4. Conclusions

An LC-MS/MS method for the quantification of caffeine and its metabolites in human plasma was validated using calibration curves prepared in plasma and in solvent. The comparison of the validation results for the linearity, precision and accuracy were similar for all molecules between the two calibration methods. The use of internal standards was fundamental for the similar results obtained for the two calibration methods since they account for the losses occurring during plasma sample processing. The chromatographic separation between paraxanthine and theophylline can be further improved in case automatic peak integration results in erroneous area calculations, however the results of this study showed that the validation parameters were acceptable.

In conclusion, whenever it is not feasible to have caffeine-free human plasma, the calibration curves in solvent can be used to quantify caffeine and its metabolites with acceptable accuracy and precision values. Moreover, it is recommended to use a certified reference material (CRM) in the appropriate matrix or, if not available, an in-house reference material (in-house RM) as a quality control (QC) for both intra- and inter-laboratory assays.

## Figures and Tables

**Table 1:** Linear regression parameters using the weighting factor 1/x^2^ for the calibration curves of caffeine, theobromine, theophylline, and paraxanthine prepared in solvent and plasma for five non-consecutive days.

Analyte	Matrix	Slope ^(1)^	Y-Intercept ^(1)^	R^2^ ^(1)^	LOD ^(3)^ (ng/mL)	LOQ ^(3)^ (ng/mL)
**Caffeine**	Plasma	0.0015 ± 0.0002	0.0010 ± 0.0004	0.9985 ± 0.0013	1.21	3.68
	Solvent	0.0016 ± 0.0002	0.0007 ± 0.0003	0.9983 ± 0.0005	0.92	2.79
	*p*-value ^(2)^	0.631	0.293			
**Theobromine**	Plasma	0.0028 ± 0.0011	0.0030 ± 0.0031	0.9984 ± 0.0016	1.39	4.22
	Solvent	0.0030 ± 0.0012	0.0006 ± 0.0008	0.9990 ± 0.0007	1.38	4.19
	*p*-value ^(2)^	0.816	0.13			
**Theophylline**	Plasma	0.0011 ± 0.0001	0.0005 ± 0.0002	0.9943 ± 0.0008	1.95	5.91
	Solvent	0.0010 ± 0.0001	0.0005 ± 0.0002	0.9957 ± 0.0028	1.85	5.61
	*p*-value ^(2)^	0.260	0.791			
**Paraxanthine**	Plasma	0.00022 ± 0.00004	0.00041 ± 0.00038	0.9932 ± 0.0088	1.32	3.99
	Solvent	0.00024 ± 0.00003	0.00001 ± 0.00006	0.9947 ± 0.0024	1.47	4.46
	*p*-value ^(2)^	0.430	0.052			

^(1)^ Calculated mean ± standard deviation (SD) for the five days. ^(2)^ Independent samples t-test applied (95% confidence; *p* < 0.05) to compare the slopes and the Y-intercepts between solvent and plasma calibration curves. ^(3)^ Limits of detection and quantification (LOD and LOQ) determination based on the standard deviation of the regression line and the slope of the calibration curve.

**Table 2:** Inter-day precision and accuracy for caffeine, theobromine, theophylline, and paraxanthine determined in calibration curves prepared in solvent and plasma. Results for the precision study are expressed as the % of the coefficient of variation (CV) and for accuracy as the % of the relative error (RE).

	**Caffeine (Solvent)**		**Caffeine (Plasma)**	
**Quality Control**	**Precision (CV%)**	**Accuracy (RE %)**	**Precision (CV%)**	**Accuracy (RE %)**
**Low**	3.3	16.4	3.4	17.4
**Medium**	2.1	0.1	2.1	3.2
**High**	1.6	−2.6	1.6	0.7
	**Theobromine (Solvent)**		**Theobromine (Plasma)**	
**Quality Control**	Precision (CV%)	Accuracy (RE %)	Precision (CV%)	Accuracy (RE %)
**Low**	7.3	12.8	8.4	7.5
**Medium**	6.6	−6.6	6.8	−2.2
**High**	6.5	−6.1	6.4	0.1
	**Theophylline (Solvent)**		**Theophylline (Plasma)**	
**Quality Control**	Precision (CV%)	Accuracy (RE %)	Precision (CV%)	Accuracy (RE %)
**Low**	3.5	21.3	3.3	11.4
**Medium**	3.0	7.1	3.0	−2.0
**High**	3.4	2.3	3.4	−6.3
	**Paraxanthine (Solvent)**		**Paraxanthine (Plasma)**	
**Quality Control**	Precision (CV%)	Accuracy (RE %)	Precision (CV%)	Accuracy (RE %)
**Low**	4.8	18.1	7.3	−8.1
**Medium**	5.5	−4.1	5.9	−1.2
**High**	8.0	−7.8	8.0	0.4

**Table 3:** Extraction efficiency (EE) and matrix effect (ME) results for caffeine, theobromine, theophylline, paraxanthine and the internal standards ^13^C_3_-caffeine and theobromine-d_6_. The evaluation was performed for three different concentration levels, the same used as quality control samples.

**Molecule**	**Parameter**	**Low**		**Medium**		**High**	
		**Mean**	**%CV**	**Mean**	**%CV**	**Mean**	**%CV**
Caffeine	EE (%)	72.9	9.0	79.1	1.7	78.9	5.5
	ME (%)	112.3	14.8	99.1	7.1	101.4	1.3
Theobromine	EE (%)	84.4	14.5	88.7	4.6	91.7	1.6
	ME (%)	116.4	21.4	94.1	8.9	93.8	5.7
Theophylline	EE (%)	84.9	18.3	86.3	1.7	90.3	2.1
	ME (%)	103.4	16.0	98.0	4.8	94.2	8.6
Paraxanthine	EE (%)	85.6	2.6	98.2	14.6	92.5	16.0
	ME (%)	117.9	27.1	93.9	7.9	101.3	16.6
		**Mean**			**%CV**		
^13^C_3_-Caffeine	EE (%)	77.7			10.1		
	ME (%)	107.4			10.3		
Theobromine-d_6_	EE (%)	89.1			15.6		
	ME (%)	104.1			14.6		

**Table 4:** Caffeine, theobromine, theophylline and paraxanthine quantification by LC-MS/MS in plasma human samples.

	Consumption (mg/day)		Absolute Quantification (ng/mL)			
Sample Code	Caffeine	Theobromine	Caffeine	Theobromine	Theophylline	Paraxanthine
1	0.0	0.0	<LOQ	<LOQ	ND	7.6
2	0.6	5.0	<LOQ	<LOQ	ND	<LOQ
3	27.1	24.6	13.0	26.3	<LOQ	123.0
4	51.7	63.8	19.3	125.8	10.8	180.0
5	52.4	5.0	9.6	47.8	<LOQ	112.1
6	53.2	75.0	<LOQ	142.0	ND	11.2
7	57.9	0.0	190.0	378.8	53.2	1014.8
8	100.0	0.0	9.7	93.2	< LOQ	23.0

<LOQ—below limit of quantification; ND—not detected.

**Table 5:** Multiple reaction monitoring (MRM) transitions of the data acquisition method for caffeine, theobromine, theophylline and paraxanthine, and the internal standards ^13^C_3_-caffeine and theobromine-d_6_.

Name	Q1	Q3	RT (min)	CE	CXP	DP
Theophylline & Paraxanthine	181.1	124.1	4.7	27	10	90
Theobromine	181.1	138.1	3.8	25	10	66
Theobromine-d_6_	187.3	144.2	3.8	25	10	91
Caffeine	195.2	138.0	6.2	27	8	71
^13^C_3_-Caffeine	198.2	140.2	6.2	27	10	61

Compound dependent parameters are described for the collision energy (CE), collision cell exit potential (CXP) and declustering potential (DP).

## Supplementary Materials

The Supplementary Materials are available online.
