# Supplementary material for: Validation of an LC-MS/MS Method for the Quantification of Caffeine and Theobromine Using Non-Matched Matrix Calibration Curve
Source: Molecules. 2019 Aug 7;24(16):2863. doi: 10.3390/molecules24162863 (PMC6720522; doi:10.3390/molecules24162863)
Supplement: Supplementary file 1 [file molecules-24-02863-s001.pdf]

Supplementary Material

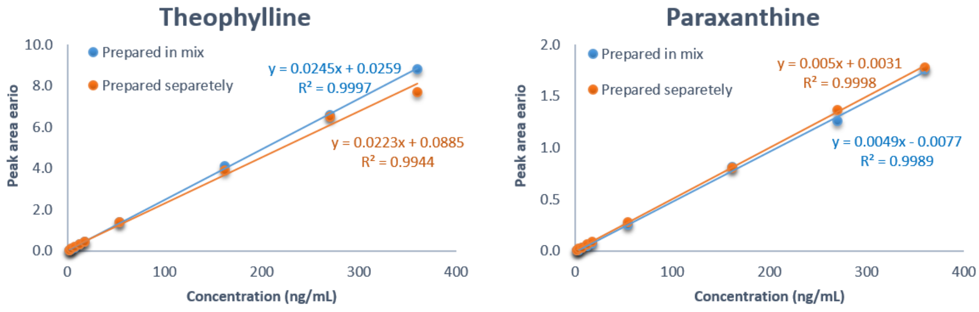

Figure S1. Comparison between the calibration curves of theophylline and paraxanthine when the two compounds were present in a mixture (blue) or were prepared separately (red). Calibrators were prepared in solvent between the range 1.8 – 360 ng/mL.

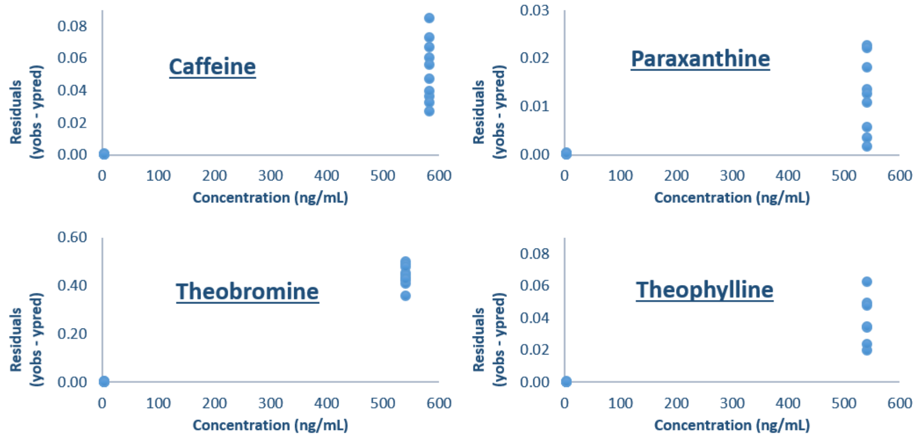

Figure S2. Homoscedasticity test for caffeine, paraxanthine, theobromine, and theophylline. Ten calibrators were analyzed for the lowest concentration and ten for the highest points of the calibration curves.

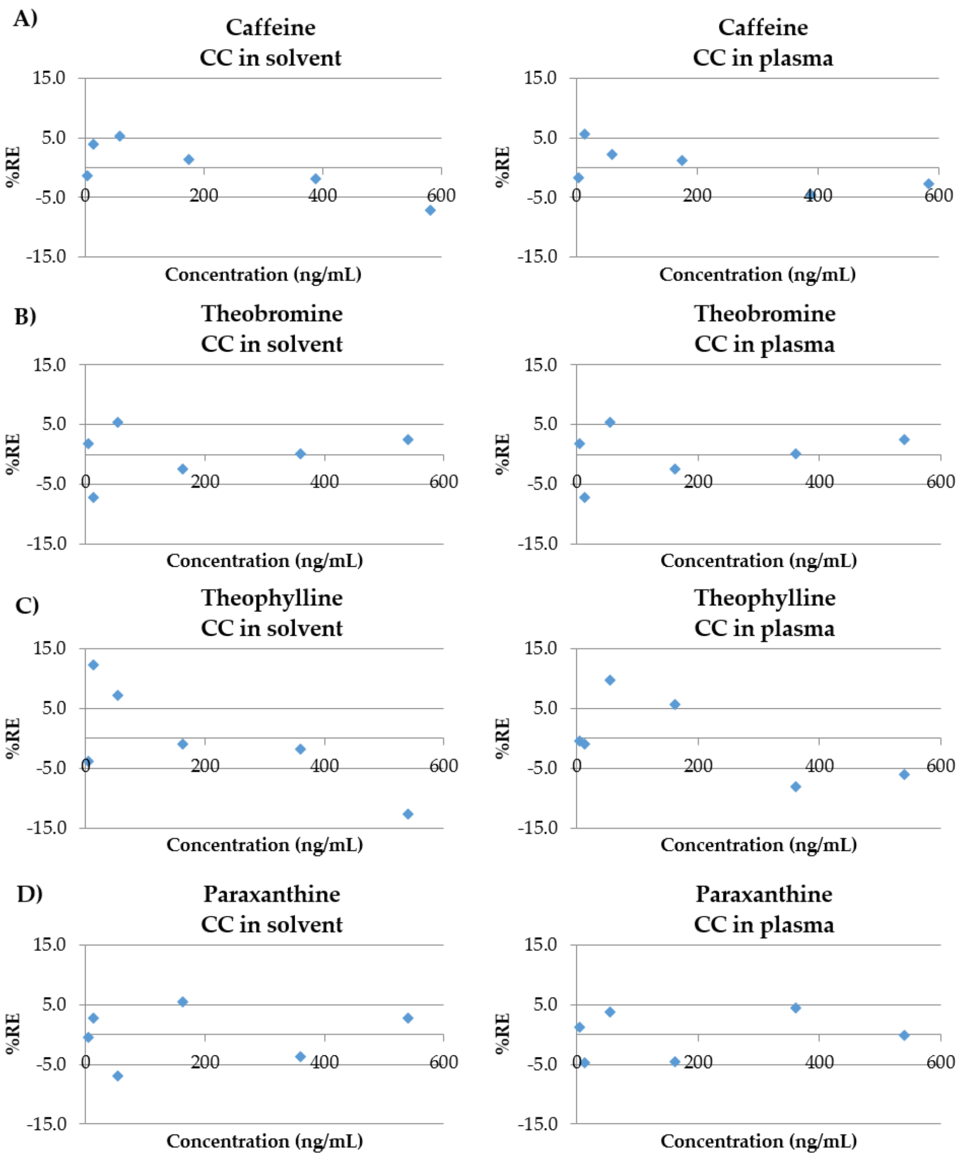

Figure S3. Percentage of relative error (%RE) versus concentration obtained for each calibration curve using the $1/x^2$ as weighting factor. The criteria of acceptance were for 1st calibrant ≤ 20% and other calibrants ≤ 15%.

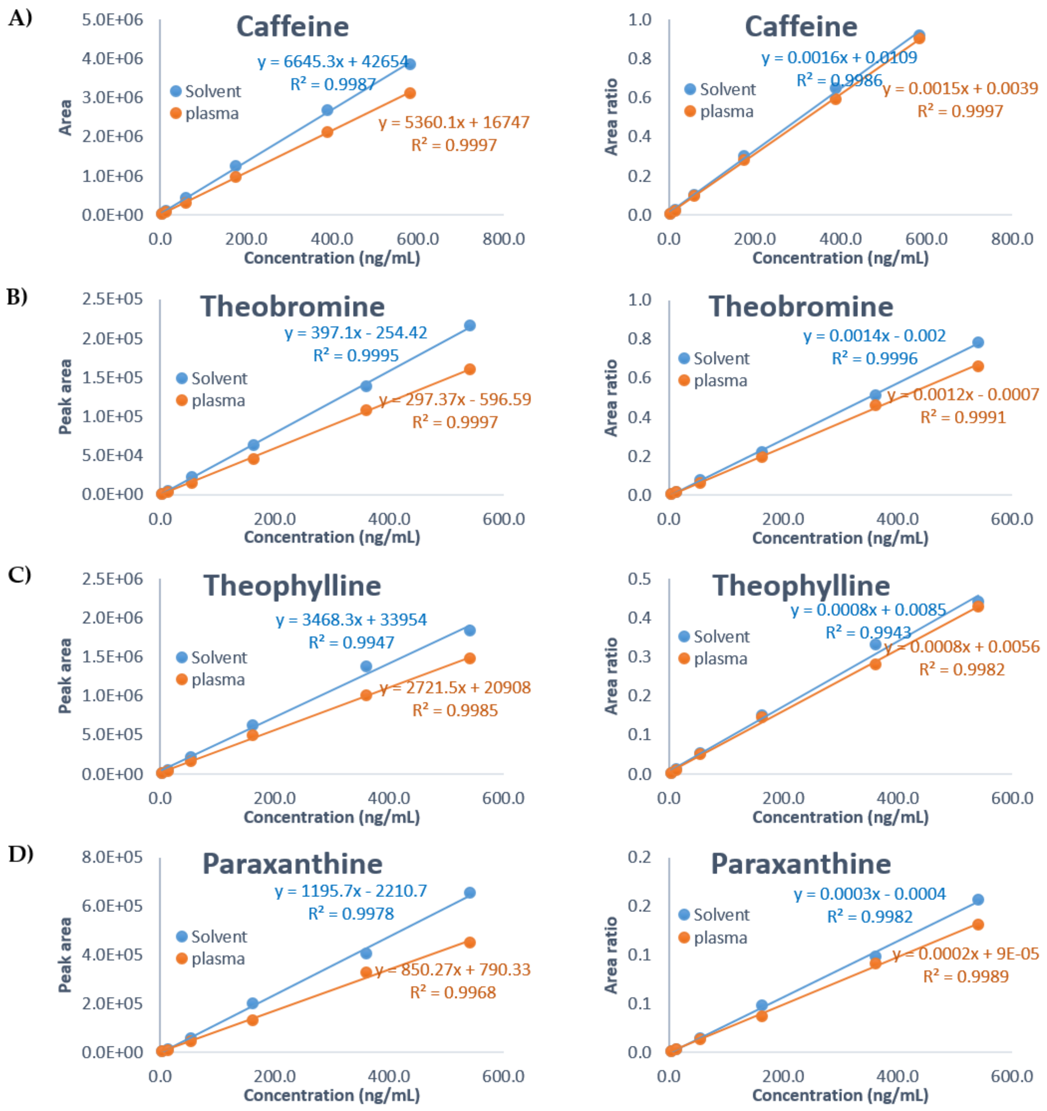

Figure S4. Calibration curves for (A) caffeine, (B) theobromine, (C) theophylline and (D) paraxanthine prepared in solvent and plasma. For each molecule and matrix, peak areas and peak area ratios were plotted against concentration.

Table S1: Selection of the best weighting factor for the regression model. Calibration curves prepared in solvent were generated for each weighting factor and the sums of the relative errors (%RE) and coefficients of determination (R^2) were calculated for each regression model.

	Caffeine		Theobromine		Theophylline		Paraxanthine	
Concentration	∑∣%RE∣	Mean R^2	∑∣%RE∣	Mean R^2	∑∣%RE∣	Mean R^2	∑∣%RE∣	Mean R^2
$1/x^0$ (OLS)	859.3	0.9986	232.2	0.9996	992.3	0.9978	604.3	0.9949
$1/x$ (WLS)	152.4	0.9987	75.7	0.9996	215.7	0.9978	173.1	0.9960
$1/x^2$ (WLS)	86.0	0.9983	63.0	0.9990	134.1	0.9957	155.2	0.9947
$1/y$ (WLS)	156.8	0.9987	76.5	0.9996	222.0	0.9978	175.1	0.9958
$1/y^2$ (WLS)	88.7	0.9982	64.0	0.9990	135.8	0.9958	155.9	0.9946
$1/x^{1/2}$ (WLS)	295.4	0.9988	103.6	0.9997	372.5	0.9981	262.9	0.9961
$1/y^{1/2}$ (WLS)	304.2	0.9988	104.0	0.9997	383.8	0.9981	267.3	0.9960
$a+bx+cx^2$ (Quad)	860.5	0.9984	261.6	0.9998	816.4	0.9982	716.7	0.9965

Table S2: Selection of the best weighting factor for the regression model. Calibration curves prepared in plasma were generated for each weighting factor and the sums of the relative errors (%RE) and coefficients of determination (R^2) were calculated for each regression model.

	Caffeine		Theobromine		Theophylline		Paraxanthine	
Concentration	∑∣%RE∣	Mean R^2	∑∣%RE∣	Mean R^2	∑∣%RE∣	Mean R^2	∑∣%RE∣	Mean R^2
$1/x^0$ (OLS)	521.1	0.9994	217.8	0.9996	1563.9	0.9966	954.3	0.9930
$1/x$ (WLS)	106.1	0.9991	78.9	0.9996	243.7	0.9955	200.4	0.9939
$1/x^2$ (WLS)	76.9	0.9985	74.7	0.9984	157.6	0.9943	153.9	0.9932
$1/y$ (WLS)	110.3	0.9992	82.5	0.9996	253.0	0.9958	233.0	0.9940
$1/y^2$ (WLS)	77.4	0.9985	77.9	0.9984	159.7	0.9944	174.8	0.9925
$1/x^{1/2}$ (WLS)	212.4	0.9994	111.2	0.9997	546.3	0.9964	359.4	0.9944
$1/y^{1/2}$ (WLS)	218.8	0.9994	114.1	0.9997	555.7	0.9966	405.7	0.9942
$a+bx+cx^2$ (Quad)	499.5	0.9992	500.4	0.9996	1505.7	0.9954	1339.1	0.9944

Table S3: The ratio of the regressed concentrations in solvent and plasma calibration curves represented by the average, standard deviation (SD) and %CV.

		Cal1	Cal2	Cal3	Cal4	Cal5	Cal6
Caffeine	average	0.99	1.04	1.00	0.99	1.02	0.96
	SD	0.01	0.04	0.04	0.03	0.03	0.03
	%CV	0.84	3.39	3.51	2.75	2.57	3.52
Theobromine	average	1.00	1.00	1.03	0.99	0.98	1.00
	SD	0.02	0.08	0.06	0.02	0.03	0.02
	%CV	1.86	7.99	5.69	2.35	2.61	1.83
Theophylline	average	0.99	1.05	0.99	0.93	1.03	1.02
	SD	0.01	0.06	0.06	0.02	0.04	0.07
	%CV	1.48	5.73	6.10	1.65	3.76	6.82
Paraxanthine	average	1.00	1.01	0.93	0.99	1.07	1.03
	SD	0.03	0.10	0.09	0.13	0.20	0.09
	%CV	2.96	10.22	10.10	12.87	19.00	8.94
